# Prospective Study of Recipient Human Leukocyte Antigen (HLA) Alloimmunization Following the Use of Cold-Stored Saphenous Vein Allografts in Vascular Surgery

**DOI:** 10.3390/jcm14041224

**Published:** 2025-02-13

**Authors:** Elsa Madeleine Faure, Pascal Pedini, Caroline Bouchet, Pascal Branchereau, Catalin Cosma, Eric Picard, Christophe Picard

**Affiliations:** 1 Department of Vascular and Thoracic Surgery, Nimes University Hospital, 30900 Nimes, France; 2 UR-UM 103 IMAGINE, University of Nimes, 30900 Nimes, France; 3 Laboratoire D’immunogénétique et Histocompatibilité, Etablissement Français du Sang PACC, 13009 Marseille, Francechristophe.picard@efs.sante.fr (C.P.); 4 ADES UMR 7268, Aix Marseille University, Etablissement Français du Sang PACC, 13009 Marseille, France

**Keywords:** hemodialysis, dialysis, arteriovenous fistula, allograft, risk assessment, immunization

## Abstract

**Objectives:** The aim of this study was to assess the HLA alloreactivity of cold-stored saphenous vein allografts (CSVAs) by identifying the production of HLA donor-specific antibodies (DSAs) in the recipient. The secondary objective was to evaluate CSVA rejection-related complications, such as CSVA thrombosis and/or aneurysmal degeneration in the recipient. **Methods:** This was a single-center, prospective, experimental before-and-after study which included participants undergoing CSVA placement, either to create a vascular access (VA) for hemodialysis or to create a lower limb arterial bypass. On Day 1, before CSVA placement, total blood samples were taken for HLA typing by sequence-specific primers (SSPs) and anti-HLA antibody detection using a Luminex assay. One month after CSVA placement, a second blood sample was taken to assess the appearance of donor-specific antibodies or an increase in the level of anti-HLA antibodies. Patency of the CSVA and potential aneurysmal degeneration were evaluated at 3 and 6 months with a Doppler ultrasound checkup. **Results**: From September 2022 to November 2023, 45 patients were included (30 men, 67%; mean age: 71 ± 12 years). One month after CSVA placement, no appearance of de novo anti-HLA antibody was detected in anti-HLA antibody-negative patients at inclusion (*n* = 28). Among the patients who already had anti-HLA antibodies at inclusion (*n* = 17), no increase in anti-HLA antibody levels or appearance of de novo anti-HLA antibodies was detected. **Conclusions:** This prospective study evaluating the immunogenicity of CSVAs through the appearance of anti-HLA antibodies one month after placement demonstrates that they do not seem to induce any HLA alloreactivity. Therefore, they may be used without the risk of HLA immunization in patients awaiting organ transplantation.

## 1. Introduction

Cryopreserved arterial and venous allografts are often used in vascular surgery [1,2,3,4]. They are particularly used to create vascular access for hemodialysis (VAH) [1,5,6] and for vascular reconstruction in organ transplantation [7,8,9,10]. However, it is now well documented in the literature that these cryopreserved vascular allografts can lead to the development of donor-specific HLA antibodies in up to 90% of cases [5,11,12,13], representing a significant issue for both indications. Indeed, this practice potentially sensitizes the recipient and may reduce their chances of receiving a kidney transplant from a larger pool of donors in the future [5,7,8,9,10,11,12,13,14,15,16,17,18].

Other alternatives to cryopreserved vessels have been developed to address the issues of preservation and availability of cryopreserved allografts. Among these alternatives, cold-stored saphenous vein allografts (CSVAs) stored at 4 °C have been used since the 1990s [19,20,21,22,23]. CSVAs are preserved at 4 °C in a saline solution of sodium chloride containing antibiotics and antifungals for at least 4 weeks before use. It is assumed that the endothelium is destroyed after 3 weeks of cold preservation (4 °C), unlike cryopreservation [24], theoretically making the grafts non-immunogenic upon implantation [25,26]. The theoretical absence of auto- or alloimmune reactions associated with CSVAs would represent a major advantage compared to cryopreserved allografts, especially when used to create a VAH or as third-party donor vessels for vascular reconstruction in organ transplantation [7,8,9,10,11,12,13,14,15,16,17,18]. However, although the alloimmunogenicity of cryopreserved allografts has been widely reported, the emergence of HLA donor-specific antibodies (DSAs) following the use of CSVAs has never been studied. Indeed, this issue has been raised in publications reporting results on CSVAs [21,22].

The aim of this study was to assess the risk of alloimmunogenicity of CSVAs by identifying the emergence of donor-specific HLA antibodies (DSAs) in the recipient, one month post-CSVA placement, using the Luminex technique. The secondary objective was to evaluate the frequency of clinical events potentially associated with CSVA rejection, such as CSVA thrombosis and/or aneurysmal degeneration in the recipient at 1 and 6 months, and to compare their occurrences among patients who developed DSA and those who did not.

## 2. Materials and Methods

This was an experimental before-and-after study.

### 2.1. Ethics Approval

The study protocol was approved on 9 June 2021 by the French Committee for the Protection of Persons (N° ID RCB 2021-A00847-34) and registered internally under number 21 55. This research was considered as a non-interventional research protocol involving human participants, classified as Category 3 according to French regulations. As such, the process for obtaining patients’ non-opposition, along with their information and non-opposition letters, was submitted to the French Committee for the Protection of Persons and received a favorable opinion. In accordance with this, a declaration of non-opposition was obtained from all participants.

Characteristics of patients, indications for CSVA placement, surgical technique, and clinical and biological outcomes were collected on a secured database. A data anonymization procedure was used, assigning a code to each individual. Only the code was entered into the computer database. Access to correspondence files required identification and access rights, which were managed by the department of information systems at Nîmes University Hospital. The biological samples were identified using a label with only the patient code and collection date.

All patients included received oral and written information about the protocol during their preoperative consultation, and only those who were unopposed to participating in the study were included.

### 2.2. Study Population

The inclusion criteria were defined as any adult volunteer undergoing CSVA placement at Nîmes University Hospital. Inclusion criteria for the use of CSVA were as defined by the ESVS 2018 guidelines [27], i.e., for VAH when autogenous options in both arms were exhausted due to poor venous capital (i.e., minimum internal vessel diameter for cephalic vein < 2 mm and for brachiocephalic and brachiobasilic arterio-venous fistulas (AVFs), a minimum venous diameter < 3 mm in both arms), thrombosis of the previous access and/or a delayed maturation defined as a venous access that is unusable after more than 6 months despite radiological or surgical intervention without other options for creating an autogenous AVF.

We also included patients receiving CSVAs for a lower limb arterial bypass in the absence of autologous saphenous vein in the context of critical ischemia associated with a risk of infection (either to revascularize diabetic foot ulcers or in cases of infected prosthesis explantation). In these cases, we used CSVAs rather than prosthetic material.

The exclusion criteria were subjects refusing to participate in the study, subjects undergoing emergency surgery without prior consultation with the surgeon, subjects who had already received a venous CSVA, subjects with active pregnancy or childbirth within the last 6 months, patients under 18 years old or under guardianship/curatorship, and patients unable to return for a check-up at one month.

### 2.3. Graft Characteristics

All cold-stored allografts used in the study were obtained from Bioprotec^®^ Inc. (Bioprotec, St. Priest, France), a human tissue bank which collects, conditions and distributes CSVAs. The saphenous vein allograft itself is collected from voluntary donors during varicose vein stripping procedures or multi-organ retrieval. The grafts are stored at 4 °C in a saline solution containing broad spectrum bactericidal antibiotics for 3 weeks. The diameter of the ducts must be between 3 and 10 mm. The technical preparation is performed by the manufacturer and consists of selecting healthy segments of veins (i.e., exclusion of dilated varicose segments), ligaturing venous collaterals, repairing micro leaks and anastomosis of 2 to 4 vein segments with monofilament polypropylene 7- or 8-0. When used electively, the surgeon orders the length and diameter of the graft required for the procedure from the manufacturer a few days beforehand (minimum 2 days).

**Figure 1:**
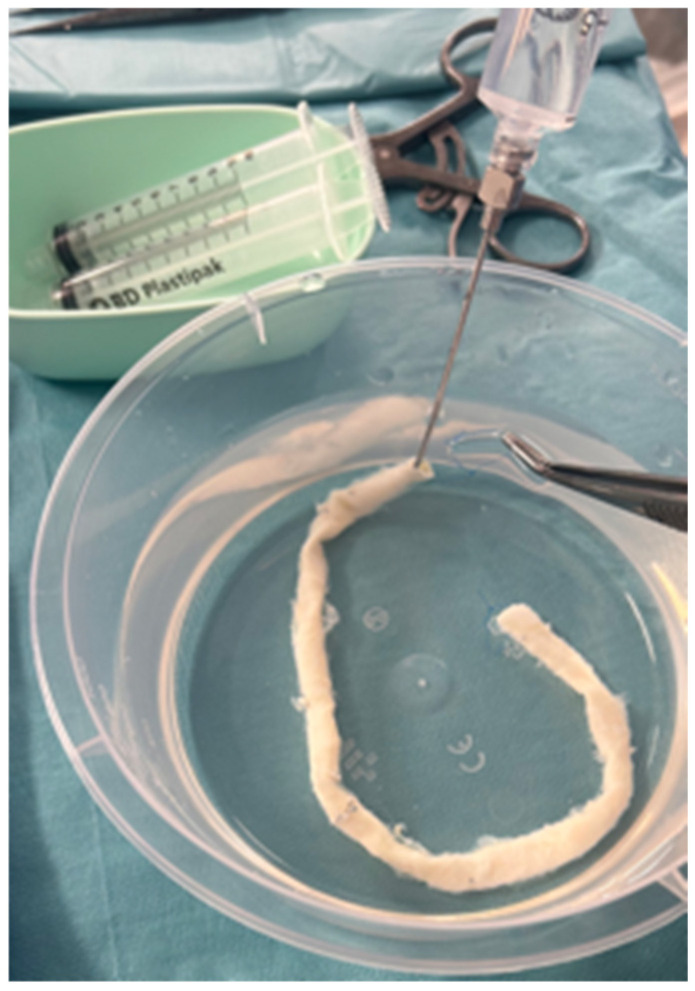
Cold-stored venous allograft is flushed/rinsed in a cup of heparinized serum under sterile conditions after removal from its preservation solution by the surgical assistant.

During the surgical procedure, the CSVAs are removed from their preservation solution by the surgical assistant and rinsed/flushed in a cup of heparinized serum under sterile conditions (Figure 1). They can then be implanted, most often by performing end-to-side anastomosis with Prolen 6-0 or 5-0.

The characteristics of the CSVAs used during the study inclusion period, including vein type, length, diameter, type of harvesting (alive or deceased donor), donor gender and age, as well as the time between vein harvesting and CSVA placement, were collected.

### 2.4. Protocol Procedure

#### 2.4.1. Day 1

Patients were included 24 h prior to CSVA placement. During the preoperative assessment, two 5 mL blood samples (one dry tube and one EDTA tube) were taken and sent to the Blood Transfusion Center (EFS) at 4° C for HLA typing and anti-HLA antibody detection. The search for anti-HLA antibodies was systematic to interpret HLA specificities.

#### 2.4.2. Perioperative Sampling on Day 0

During the surgical procedure, a sample of CSVA was collected and sent to the French blood center in Marseille so that the donor’s HLA system could be analyzed in the event that anti-HLA antibodies should appear in the recipient at one month post-operation (DSA determination). In the latter case, additionally, the recipient would be HLA typed (interpretation of specificities).

#### 2.4.3. One-Month Follow-Up

During the one-month postoperative consultation, a 5 ml blood sample (dry tube) was taken and sent for anti-HLA antibody detection. The level of anti-HLA antibodies in this sample was compared to the preoperative sample. If an appearance or increase in anti-HLA antibody levels was detected in this second sample, characterization of the anti-HLA antibodies was performed on the patient’s sample, as well as HLA system analysis of the donor sample taken from the CSVA. A questionnaire detailing any immunizing events occurring in the previous month, such as transfusion, was conducted during the consultation, along with a clinical examination of the CSVA by visual examination of the postoperative healing and palpation of the peripheral pulses and/or thrill. A Doppler ultrasound checkup was made on the same day.

#### 2.4.4. Six-Month Follow-Up

A clinical examination and Doppler ultrasound checkup were made at the six-month postoperative consultation. Any events or reinterventions on the CSVA bypass that had occurred in the meantime were recorded.

### 2.5. HLA Typing and Anti-HLA Antibody Detection

The HLA typing of recipient and donor was obtained using the SSP FluoVista technique v.1.8.1.0 (Innotrain^®^, Taunus, Germany) after extracting DNA from frozen blood (EDTA tube) and frozen tissue, respectively. DNA was extracted from blood using the LabTurbo^®^ automated system and, from tissue, using a manual technique (QIAmp DNA tissue, QIAGEN, Hilden, Germany) following overnight digestion with nucleases.

The Luminex technique (LABScreen™ Mixed, One lambda, West Hills, CA, USA) was used to screen for anti-HLA antibodies. Specificities of anti-HLA antibodies were determined using Luminex single antigen (LSA) tests for class I and class II (LABScreen™ Single Antigen, One Lambda) on screening positive sera. ‘Single Antigen’ commercial reagents were used according to the manufacturer’s instructions, making it possible to identify specificities targeted by the antibodies. Briefly, a small volume of serum samples was incubated with the beads. After washing, the sensitized beads were detected using a human anti-IgG antibody conjugated with phycoerythrin. The results were read with the Labscan^®^ 200 device, then validated and interpreted with Fusion interpretation software (ThermoFisher Scientific v4.4, Waltham, MA, USA). For each serum, the specificities of each antibody were identified according to the positive beads in the panel, determined by Mean Fluorescence intensity (MFI) > 1000. All positive results were interpreted based on the donor’s and recipient’s HLA typing to determine the presence or absence of DSAs. To monitor the changes in the anti-HLA antibody profile between 24 h prior to CSVA placement and one-month follow-up, when positive beads were identified, the panel reactive antibodies (PRAs) were calculated as a percentage by dividing the number of reactive beads by the total number of beads in the panel and multiplying by 100. The results and their interpretations were then recorded in the medical–technical software available in the laboratory (Inlog v.7.1.0.5).

### 2.6. Data Analysis

Statistical analyses were performed using SPSS (Released 2020. SPSS for MacOS, Version 27.0., IBM Corp in Armonk, NY, USA). A descriptive analysis was conducted on the entire study population regarding sociodemographic, clinical and paraclinical variables available at inclusion. Finally, a Calculated Panel Reactive Antibody (CPRA) score was estimated to determine the percentage of donors from whose organs the patient would be incompatible. Qualitative variables were summarized as counts and percentages, and quantitative variables were expressed as means (±standard deviation) or medians (interquartile range) for normally and non-normally distributed data, respectively. The secondary objective was to compare the occurrence of complications between two groups, defined according to whether DSAs had emerged at one month or not. However, in this study, no emergence of DSAs was detected, making it impossible to form the two groups required for a comparative analysis. Consequently, no comparative statistical analysis could be made for this secondary objective. Nonetheless, the descriptive results of complications observed were recorded for the entire cohort.

## 3. Results

From September 2022 to November 2023, 45 patients were included (30 men, 67%; mean age: 71 ± 12 years). CSVAs were placed in the context of chronic or critical ischemia of the lower limbs in twenty-nine patients (64%), including three patients for whom revascularization was performed after explantation of an infected prosthetic graft, and sixteen patients requiring creation of a VA for hemodialysis (36%). The characteristics of all 45 patients included are reported in Table 1.

The average length of the bypass graft was 34 cm (±14 cm). During the inclusion period, the CSVAs were saphenous veins harvested from living donors in 93% of cases and deceased donors in 7%, and 59% were harvested from men (mean age of the donor: 56 years). The mean storage time between harvesting and CSVA placement was 16 weeks (±6 weeks). The characteristics of CSVAs are detailed in Table 2.

In the 24 hours prior to CSVA sampling, no anti-HLA antibodies were detected in 28 patients (62%), whereas preoperative anti-HLA antibodies were detected in 17 patients (38%). Regarding patients with anti-HLA antibodies at 24 hrs prior to CSVA placement, ten were males with a history of transfusion, and seven were females with a history of pregnancy in all cases and history of transfusion in five cases. The type of anti-HLA antibodies and median percentage of panel reactive antibodies detected on Day 1 and at one month are detailed in Table 3.

The descriptive results of screening for anti-HLA antibodies at one month, as well as complications and patency rates at one- and six-month follow-up, are reported in Table 4.

At one-month follow-up, no appearance of de novo anti-HLA antibodies was detected in anti-HLA antibody-negative patients at 24 h prior to CSVA placement. Among patients who already had anti-HLA antibodies on Day 1, no increase in anti-HLA antibody levels nor appearance of de novo anti-HLA antibodies was detected at one month.

After one month, primary patency was 98% (*n* = 44/45). One patient had a successful thrombectomy (secondary patency 100%). Three patients had a surgical site infection with a favorable evolution under antibiotic therapy and directed healing. One patient had a false aneurysm at the distal anastomosis of the femoropopliteal bypass.

Between 24 h prior to CSVA placement and one-month follow-up, four patients were transfused. No other potentially immunizing events were reported.

After six months’ follow-up, primary patency was 78% (*n* = 35/45) and secondary patency was 89% (*n* = 40/45). Two patients underwent successful thrombectomy, and two others underwent angioplasty: one for stenosis of the distal venous anastomosis of his humero-axillary bypass and the other for stenosis of the iliac artery upstream of the bypass. Five patients had lost their bypass; two patients underwent transfemoral amputation due to an unfavorable progression of their foot healing despite revascularization, one patient had closure of his humero-axillary bypass due to a vascular access-induced limb ischemia in the upper access, and two other patients had failure of their femoro-tibial bypass thrombectomy. No new aneurysmal degeneration was detected at six-month follow-up.

As CSVA did not induce HLA alloreactivity in our study, we were unable to statistically analyze the likelihood of complications based on the development of DSAs at one month in the recipient.

## 4. Discussion

We report the first study to evaluate the immunological response after placement of CSVA preserved for at least 4 weeks before use.

There have already been reports on the immune response monitored after an arterial and/or venous allograft bypass, but those studies focus on cryopreserved allografts [5,11,12,13], whereas ours evaluates the immune response after venous allografts preserved at 4 °C. Our study also presents the results on monitoring the immune response after an arterial and/or venous bypass in which HLA antibodies were determined using the Luminex method, which provides detailed, antigen-specific information [5,11,12,13].

In this study, no detectable HLA allo-reactivity was observed after implanting CSVAs. By comparison, an HLA immunization rate of 60% to 90% has been reported after cryopreserved venous allograft implantation [5,7,8,9,10,11,12,13,14,15,16,17] and a 20% HLA immunization rate between 2 weeks and 3 months after blood transfusion [28,29].

Despite our study’s small sample size, its results are significant because, if confirmed in a larger cohort, this technique could provide a valuable alternative to cryopreserved allografts for VAH, as well as for vascular reconstruction in organ transplantations. Indeed, third-party vascular allografts are an invaluable resource for kidney and pancreas transplants when vascular reconstruction is required and additional vessels from the organ donor are unavailable [9]. Another option for vascular reconstruction is to use synthetic grafts which may carry a higher risk of thrombosis or infection [9,22].

In our study, CSVAs appear to be associated with lower immunogenicity than cryopreserved allografts and would therefore increase the chances of receiving a future transplant compared to cryopreserved vascular allografts. However, these results are tempered by the small cohort size of our single-institution analysis, and further studies to confirm them are required.

As reported in the literature, the problem of immune reaction after allograft placement is crucial, especially when these grafts are placed in patients likely to receive an organ transplant [5,11,12]. Indeed, recipient immunization is a significant issue in organ transplantation, as it leads to particular difficulties in selecting a donor and, more importantly, exposes the recipient to a greater risk of graft rejection [14,15,16]. Antibody-mediated rejection is the leading cause of graft loss after kidney transplants [30,31,32]. Recently, Sadaghianloo et al. reported the case of a patient awaiting renal transplantation who underwent the placement of a cryopreserved arterial allograft as an interposition on an infected VA for hemodialysis, subsequently developing class I and II anti-HLA antibodies. The patient was successfully transplanted but was considered at a high risk of rejection and required an increase in immunosuppressive treatment [18]. 

The development of DSAs due to cryopreserved arterial and/or venous allografts is well documented [5,11,12,13]. Cryopreservation preserves the endothelium where the cells presenting HLA molecules are located [24,33]. In the context of cold preservation (preservation at 4 °C), unlike cryopreservation [24], studies have already shown that preservation solutions impact endothelium integrity [25,26,34,35]. Compared with cryopreserved vascular allografts, the absence of HLA allo-reactivity with CSVAs is probably explained by the destruction of the endothelium, where cells presenting HLA molecules are located, during the 4 °C preservation period. Bioprotec recommends cold preservation of CSVAs for at least 4 weeks before transplantation, to sufficiently alter the endothelium. In our study, the mean preservation period was 16 weeks (±6 weeks) before implantation, which is relatively short considering they can be preserved for up to 12 months. Our study demonstrates that this timeframe is sufficient for CSVAs to be transplanted without any risk of HLA allo-reactivity. A storage period of less than 4 weeks for CSVAs before use may be insufficient to cause complete endothelial alteration and probably increases the risk of recipient immunization.

Nevertheless, although the absence of endothelium may confer an advantage over cryopreserved allografts in terms of allergenic reactivity, it may also prove to be a disadvantage in terms of permeability. Indeed, some authors consider that destruction of the venous endothelium is a risk factor for thrombosis [34,36]. Regarding CSVAs, several studies have already demonstrated that their patency is acceptable for both lower limb arterial bypasses and hemodialysis vascular access [19,20,21,22,23,37].

In a meta-analysis on infrapopliteal arterial bypasses using allografts, Albers et al. [38] reported that, in terms of 4-year patency, CSVAs showed the best outcomes, followed by cryopreserved arteries, umbilical cord veins and cryopreserved veins. However, they also suggested that there are probably no significant differences between the various types of allografts, as the confidence intervals for their pooled estimates overlapped too extensively [38]. Furthermore, compared to cryopreserved allografts, CSVAs are more readily available and their preservation and use are simplified, as they are prepared and tested in advance by the laboratory and only require refrigerator storage. Ultimately, with a similar patency rate and lower immunogenicity, CSVAs should probably be used preferentially for organ transplantation or in patients eligible for future organ transplant.

In this study, we report a primary patency and a primary assisted patency rate of 97% and 100%, respectively, at one-month follow-up and a pseudo-aneurysmal evolution of 2%. Since no patients developed DSAs, we can conclude that, in the context of CSVAs, thrombosis and pseudo-aneurysmal degeneration are not related to an immune reaction. Concerning cryopreserved allografts, the association between immune response against vascular transplants and bypass degradation or reduced time for the development of new aneurysms in subsequent allografts has not been demonstrated [17].

A one-month timeframe was selected to evaluate the development of DSAs to ensure sufficient distance from the potentially immunizing event (as anti-HLA antibodies typically develop 2 to 4 weeks after such an event) but not too distant to avoid the risk of false positives due to the occurrence of other immunizing events during this period, such as transfusion or vaccination. The 1-month and 6-month timepoints were chosen to assess the onset of clinical signs of both acute and chronic cold-stored veinous allograft rejection potentially associated with the development of DSAs.

Recently, other immune mechanisms have been found to be involved in organ transplant rejection, but these were not investigated here. Such mechanisms are minor compared to the development of DSAs in the recipient in the rejection process and are unlikely to occur in recipients of vascular allografts [39]. The risk of HLA alloimmunization by endothelial cells in different organ transplants without immunosuppressive therapy is very high. In this context, given the central role of endothelial cells in modulating the allo-immune response, graft endothelium is currently a preferential target for newer immunosuppressive protocols aimed at promoting long-term graft acceptance by reducing endothelial cell immunogenicity and antigen presentation, whilst favoring their survival. Thus, the expected HLA allo-immunization and DSA detection in CSVAs was high, especially in those patients who already had HLA immunization.

This study has several limitations. It is only a single-center observational study involving the study of a series of patients from the southeast of France, grafted by a single surgical team. In this context, a center effect cannot be excluded and the comparison of results with cryopreserved arterial allografts is purely bibliographical. Although the absence of immunogenicity of CSVAs in 45 patients is reassuring regarding their use in patients potentially eligible for an organ transplant, making it possible to maintain every chance of finding a compatible kidney donor, multi-center studies involving a larger cohort are necessary to validate these findings. Moreover, we included patients receiving CSVAs for two different indications (VA for hemodialysis and chronic or critical ischemia of the lower limbs), since the aim of this study was to evaluate the immunogenicity of CSVAs rather than their patency outcomes. However, as the CSVAs used for lower limb bypass were longer, the risk of allo-immunogenicity was theoretically higher in these patients. Lastly, future research with long-term follow-up comparing patency and recipient immune response between CSVAs and cryopreserved allografts would be interesting.

## 5. Conclusions

This prospective study evaluating the immunogenicity of CSVA through the appearance of anti-HLA antibodies one month after their placement demonstrates that, unlike cryopreserved allografts, they do not seem to induce any HLA alloreactivity. Therefore, they may be used without risk of HLA immunization in patients awaiting organ transplantation.

## Figures and Tables

**Table 1:** Baseline characteristics of the 45 patients undergoing CSVA placement.

		Overall Data = 45
Women		15 (33)
Age		71±12
Diabetes mellitus		27 (60)
Body mass index, kg/m^2^		27 ±8
Hypertension		39 (87)
Active smoking		9 (20)
Pulmonary disease		7 (16)
Stroke/TIA		3 (7)
Heart failure		14 (31)
Coronary artery disease		21 (47)
ASA Score	- 2	2 (4)
	- 3	15 (33)
	- 4	28 (62)
Peripheral arterial disease		34 (76)
Active cancer		1 (2)
Prior kidney transplant		0 (0)
Immunosuppressive drugs		0 (0)
Corticosteroids		2 (4)
Anticoagulants		13 (29)

Continuous variables are reported as the mean and standard deviation; categorical variables are reported as numbers (%); TIA = transient ischemic attack, ASA Score (American Society of Anesthesiologists).

**Table 2:** Characteristics of the donors.

		Lower Limb Arterial Bypass *n* = 29	Vascular Access for Haemodialysis *n* = 16	OR (95%CI)	*p*-Value
Age, years ± SD		71 ± 10	71 ± 15		
Gender, male (%)		22 (75.9)	8 (50)	3.06 [0.71; 13.93]	0.105
CSVA characteristics					
	Living donor (%)	27 (93)	15 (94)	0.90 [0.01; 18.71]	1
	Gender, male (%)	18 (56)	10 (62.5)	0.98 [0.23; 4.06]	1
	Length, mm ± SD	42.8 ± 8.8	17.5 ± 4.5	[21.26; 29.26]	<0.001
	Time from harvesting to placement in weeks ± SD	15.9 ± 6.4	17.3 ± 6.8	[−5.67; 2.78]	0.489

Continuous variables are reported as means and standard deviations; categorical variables are reported as numbers (%). SD: standard deviation; OR: odds ratio; CI: confidence interval.

**Table 3:** Types of anti-HLA antibodies detected and percentage of panel reactive antibodies at inclusion and one month.

	Class I (mPRA%, Range Min–Max)	Class II (mPRA%, Range Min–Max)	Class I and II (Cl I mPRA%, Range Min–Max) (Cl II mPRA%, Range Min–Max)
Anti-HLA antibodies present at inclusion *n* = 17	11 (5, 1–19)	0	6 (20.5, 2–95) (14, 1–65)
Anti-HLA antibodies present at M1 *n* = 14	9 (3, 2–24)	0	5 (32, 6–94) (20, 4–64)

Anti-HLA: anti-human leukocyte antigen; Cl: class; M: month; min: minimum; max: maximum; mPRA%: median percentage of panel reactive antibodies.

**Table 4:** Descriptive results of screening for anti-HLA antibodies at one month, complications and patency rates at one-month and six-month follow-up.

		Anti-HLA Antibodies Absent at Inclusion *n* = 28 (62)		Anti-HLA Antibodies Present at Inclusion *n* = 17 (38)	
		LLA bypass *n* = 20 (71)	VAH *n* = 8 (29)	LLA bypass *n* = 9 (53)	VAH *n* = 8 (47)
**One-month follow-up**					
	Appearance of de novo anti-HLA antibody	0 (0)	0 (0)	0 (0)	0 (0)
	Increase in anti-HLA antibody levels	0 (0)	0 (0)	0 (0)	0 (0)
	Aneurysmal degeneration	1 (5)	0 (0)	0 (0)	0 (0)
	Primary patency	20 (100)	8 (100)	9 (100)	7 (87.5)
	Primary assisted patency	20 (100)	8 (100)	9 (100)	7 (87.5)
	Secondary patency	20 (100)	8 (100)	9 (100)	8 (100)
**6-month follow-up**					
	Aneurysmal degeneration	0 (0)	0 (0)	0 (0)	0 (0)
	Primary patency	18 (90)	6 (75)	6 (67)	5 (62.5)
	Primary assisted patency	19 (95)	7 (87.5)	6 (67)	5 (62.5)
	Secondary patency	19 (95)	8 (100)	6 (67)	7 (87.5)

Categorical variables are reported as numbers (%). LLA: lower limb arterial; VAH: vascular access for hemodialysis; Anti-HLA: anti-human leukocyte antigen.

## Data Availability

Data are unavailable due to privacy restriction.

## Abbreviations

The following abbreviations are used in this manuscript:

CSVA	Cold-stored saphenous vein allografts
DSA	Donor specific antibodies
VA	Vascular access
